# Mn-TAT PTD-Ngb attenuates oxidative injury by an enhanced ROS scavenging ability and the regulation of redox signaling pathway

**DOI:** 10.1038/s41598-019-56595-5

**Published:** 2019-12-27

**Authors:** Cui Zhang, Xuehui Hao, Jiaying Chang, Zhirong Geng, Zhilin Wang

**Affiliations:** State key Laboratory of Coordination Chemistry, School of Chemistry and Chemical Engineering, Collaborative Innovation Center of Advanced Microstructures, Nanjing University, Nanjing, 210023 P.R. China

**Keywords:** Bioinorganic chemistry, Mechanism of action

## Abstract

Neurological diseases have a close relationship to excessive reactive oxygen species (ROS). Neuroglobin (Ngb), an intrinsic protective factor, protected cells from hypoxic/ischemic injury. In the present, we reported a novel neuroprotective manganese porphyrin reconstituted metal protein, Mn-TAT PTD-Ngb, consisting of a HIV Tat protein transduction domain sequence (TAT PTD) attached to the N-terminal of apo-Ngb. Mn-TAT PTD-Ngb had a stronger ROS scavenging ability than that of TAT PTD-Ngb, and reduced intracellular ROS production and restored the function of the mitochondria and inhibited the mitochondria-dependent apoptosis. Besides, Mn-TAT PTD-Ngb activated the phosphoinositide-3 kinase (PI3K)/Akt signaling pathway, which up-regulated the expression of nuclear factor E2-related factor 2 (Nrf2), Heme oxygenase-1 (HO-1), superoxide dismutase (SOD), catalase (CAT). The results showed that the redox chemistry of Mn-TAT PTD-Ngb and redox regulation of multiple signaling pathways attenuated the oxidative injury.

## Introduction

Neurological diseases, such as stroke, often lead to a high rate of disability and death as well as impose a great burden on the family and society^1,2^. Accumulating evidences implicate enhanced oxidative stress, mitochondrial dysfunction, and activation of apoptosis play critical role in the propagation of neuronal injury^3–5^. Excessive ROS was closely related to activation of specific redox signaling pathways that modulated redox homeostasis^6,7^. A series of globin were reported to fight against ROS and protect neurons from mitochondrial dysfunctions^8,9^. Ngb was a newly discovered member of the globin family, and it functioned as a redox signaling protein and possessed antioxidant property towards ROS in hypoxia and ischemia^10–15^. Mitochondrion, an important organelle that acted as the redox center, was the main source of excessive ROS under oxidative injury, which ultimately led to mitochondrial ATP loss, collapsed mitochondrial membrane potential and the release of cytochrome c (Cyt c)^16^. Ngb reduced ferric Cyt c to prevent the formation of the apoptosome and activation of caspase-9 and apoptosis executor caspase-3^17,18^. PI3K/Akt pathway was an important signaling pathway to promote neuron survival, Ngb also participated in the PI3K/Akt pathway and attenuated the production of Aβ to promote neuron survival^19–22^. Up-regulating phosphorylated Akt prevented the expression of Bad, Bax and the activation of caspases cascade. Nrf2 was a master regulator of redox homeostasis, which was down-regulated by GSK-3β^23^. Phosphorylated Akt increased the phosphorylated GSK-3β, leading to up-regulated Nrf2, HO-1, SOD, CAT, the downstream target of Nrf2, acted as antioxidant defense line^23–25^ and protected cells against oxidative injury.

The redox chemistry of Ngb is determined by its inherent metal center, with a more negative potential being more desirable for redox reactions^26^. Manganese is a transition element with multiple valances and is an important constituent of SOD^27^. Mn-porphyrin showed a more negative potential and is more biologically tolerable *in vivo* compared to heme. Mn-porphyrin and its derivatives have been widely studied in neurological diseases^28–31^. In MCAO mice, MnTm4PyP is used as a highly efficient scavenger of intracellular O_2_^−^ and hydrogen peroxides (H_2_O_2_) and up-regulates intracellular antioxidant systems in neurons to promote cell survival^32^. Recently, artificial proteins have emerged as a powerful tool to design and manipulate the properties of proteins^33,34^. A series of artificial proteins of apo-myoglobin (apo-Mb), apo-hemoglobin (apo-Hb) and apo-horseradish peroxide (apo-HRP) reconstituted with different metals were reported, and some displayed ROS scavenging ability or other new properties such as nitrite reductase (NiR) activity^35^. Taniguchi reported that an artificial protein composed of apo-Mb reconstituted with Mn-porphyrin has a more negative potential than Mb. However, there has been no reporting on apo-Ngb reconstituted with Mn-porphyrin until now^36^.

A novel artificial metal protein Mn-TAT PTD-Ngb was prepared and displayed a more negative potential and enhanced ROS scavenging ability than TAT PTD-Ngb. Mn-TAT PTD-Ngb played critical roles in regulating redox signaling to attenuate oxidative injury. It maintained mitochondria function, such as restoring the mitochondrial membrane potential and inhibiting the loss of adenosine triphosphate (ATP). To promote cell survival, it prevented the activation of mitochondria-dependent apoptosis. Besides, Mn-TAT PTD-Ngb could activate PI3K/Akt pathway and promote the up-regulation of Nrf2, HO-1, which increased the expression of antioxidant defense enzymes such as SOD, CAT. However, Mn-TAT PTD-Ngb had no significant effect on relieving endoplasmic reticulum (ER) stress, which was different from the effects of Ngb. Mn-TAT PTD-Ngb may be a novel candidate in treating neurological diseases.

## Results

### Expression, purification and spectral analysis of TAT PTD-Ngb, Mn-TAT PTD-Ngb

**Figure 1:**
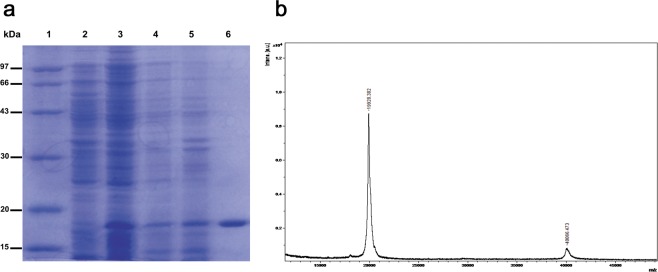
(**a**) SDS-PAGE analysis of expressed and purified TAT PTD-Ngb in *E. Coli* BL21(DE3). 1. protein marker; 2. whole lysate of *E. Coli* BL21(DE3) without IPTG; 3. lysate of *E. Coli* BL21(DE3) induced by IPTG (0.1 mg/ml); 4. precipitate of the whole lysate of *E. Coli* BL21(DE3) induced by IPTG; 5. supernatant of the whole lysate of *E. Coli* BL21(DE3) induced by IPTG; 6. purified fusion protein. (**b**) MALDI-TOF-MS spectrum analysis of TAT PTD-Ngb.

**Figure 2:**
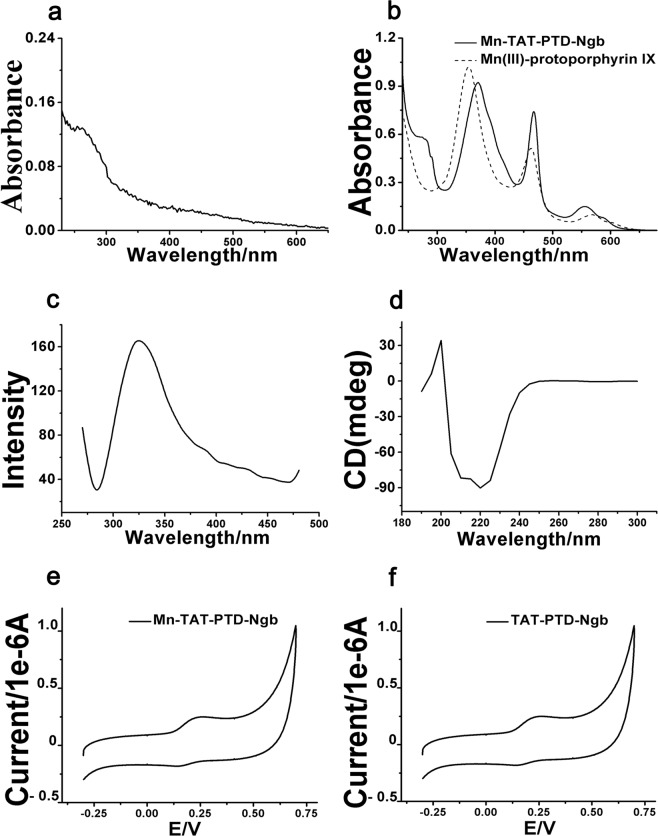
Spectral analysis of Mn-TAT PTD-Ngb. (**a**) UV-Vis spectrum of apo-TAT PTD-Ngb. (**b)** UV-Vis spectrum of Mn(III)-protoporphyrin IX, Mn-TAT PTD-Ngb. (**c**) Fluorescence spectrum of Mn-TAT PTD-Ngb. (**d)** Circular dichroism spectrum of Mn-TAT PTD-Ngb. (**e,f**) Cyclic voltammogram of TAT PTD-Ngb, Mn-TATPTD-Ngb at pH 7.4 in 50 mM Na_2_HPO_4_-NaH_2_PO_4_. The gold electrode was modified with 10 mM cysteine and mercaptoethanol. The reference electrode was Ag|AgCl|3 M KCl and the scan rate was 100 mV.s^−1^.

The recombinant plasmid contained the sequences of TAT PTD, a His-tag and Ngb (Fig. S1). The fusion protein included a TAT PTD sequence (YGRKKRRQRRR), which helped the fusion protein move across the cell membrane. A 6 × His tag was added to facilitate purification and identification of the fusion proteins. The total protein expression levels of TAT PTD-Ngb reached the highest point when the concentration of IPTG was 0.1 mg/ml. The fusion protein was analyzed by sodium dodecyl sulfate-polyacrylamide gel electrophoresis (SDS-PAGE) and MALDI-TOF-MS (Fig. 1). SDS-PAGE analysis showed that the expression of TAT PTD-Ngb in the *E. coli*BL21(DE3)plysS cells was mainly in the soluble fraction (Fig. 1a). The molecular weight of the obtained fusion protein was 20 kDa (Fig. 1b). The UV-Vis spectra of TAT PTD-Ngb, apo-TAT PTD-Ngb and Mn-TAT PTD-Ngb are shown in Fig. S2a and Fig. 2a,b, respectively. TAT PTD-Ngb showed absorbance at 280 nm, 413 nm and 500–600 nm, which was similar to wild type-Ngb. Mn-TAT PTD-Ngb showed the characteristic absorbance of protein at 280 nm and Soret band absorbance of Mn(III)-protoporphyrin IX at 374 nm, as well as an additional p–d transition band at 468 nm and a β band at 500–600 nm, which were similar to those of manganese(III)-reconstituted myoglobin, Cyt c, and hemoglobin^34^. In the fluorescence spectrum, TAT PTD-Ngb showed an emission wavelength of 340 nm at excitation at 280 nm, while Mn-TAT PTD-Ngb emitted at 330 nm, suggesting that the reconstituted metal center had no significant effect on fluorescence spectrum (Fig. S2b,c). The conformational changes of the reconstituted metal protein and TAT PTD-Ngb were assayed by circular dichroism (CD) spectroscopy. Both of the proteins showed two negative peaks at 220 nm and 208 nm, which suggested α-helix structure. The secondary structure was elucidated using Jwsse32 software with reference CD Yang. jwr. The data indicated that there was no significant difference between the secondary structures of Mn-TAT PTD-Ngb and TAT-PTD-Ngb (Figs. 2d and S2c and Table 1). The electrochemical redox behaviors of the two fusion proteins were measured by cyclic voltammetry (CV). The CV data showed that Mn-TAT PTD-Ngb had a more negative potential than TAT PTD-Ngb (Fig. 2e,f).

**Table 1:** Secondary structure analysis of TAT-PTD-Ngb and Mn-TAT PTD-Ngb.

Parameters	TAT PTD-Ngb	Mn TAT PTD-Ngb
Helix	15.16 ± 0.011	13.88 ± 0.119
Beta	9.513 ± 0.019	12.48 ± 0.031
Turn	16.43 ± 0.013	13.53 ± 0.017
Radom	17.22 ± 0.025	14.92 ± 0.016

### Translocation of Mn-TAT PTD-Ngb into PC12 cells

PC12 cells were incubated with Mn-TAT PTD-Ngb for 6 h at the concentration of 0.1 μM, 0.5 μM, 1.0 μM, 1.5 μM, respectively. The intracellular Mn-TAT PTD-Ngb levels of PC12 cells were detected by Western blot. Mn-TAT PTD-Ngb delivered into PC12 cells in a dose-dependent manner (Fig. S3a).

To investigate the effect of incubation time on the translocation of Mn-TAT PTD-Ngb into PC12 cells, 1.0 μM Mn-TAT PTD-Ngb was incubated with Mn-TAT PTD-Ngb for 2 h, 6 h, 24 h, 36 h, respectively. The result indicated that the concentration of the intracellular Mn-TAT PTD-Ngb peaked at the incubation time of 6 h and decreased at the incubation of 24–36 h (Fig. S3b). The transduction of Mn-TAT PTD-Ngb into PC12 cells followed a time-dependent manner.

### Mn-TAT PTD-Ngb attenuates H_2_O_2_-induced ROS levels

#### Mn-TAT PTD-Ngb inhibits ROS production

**Figure 3:**
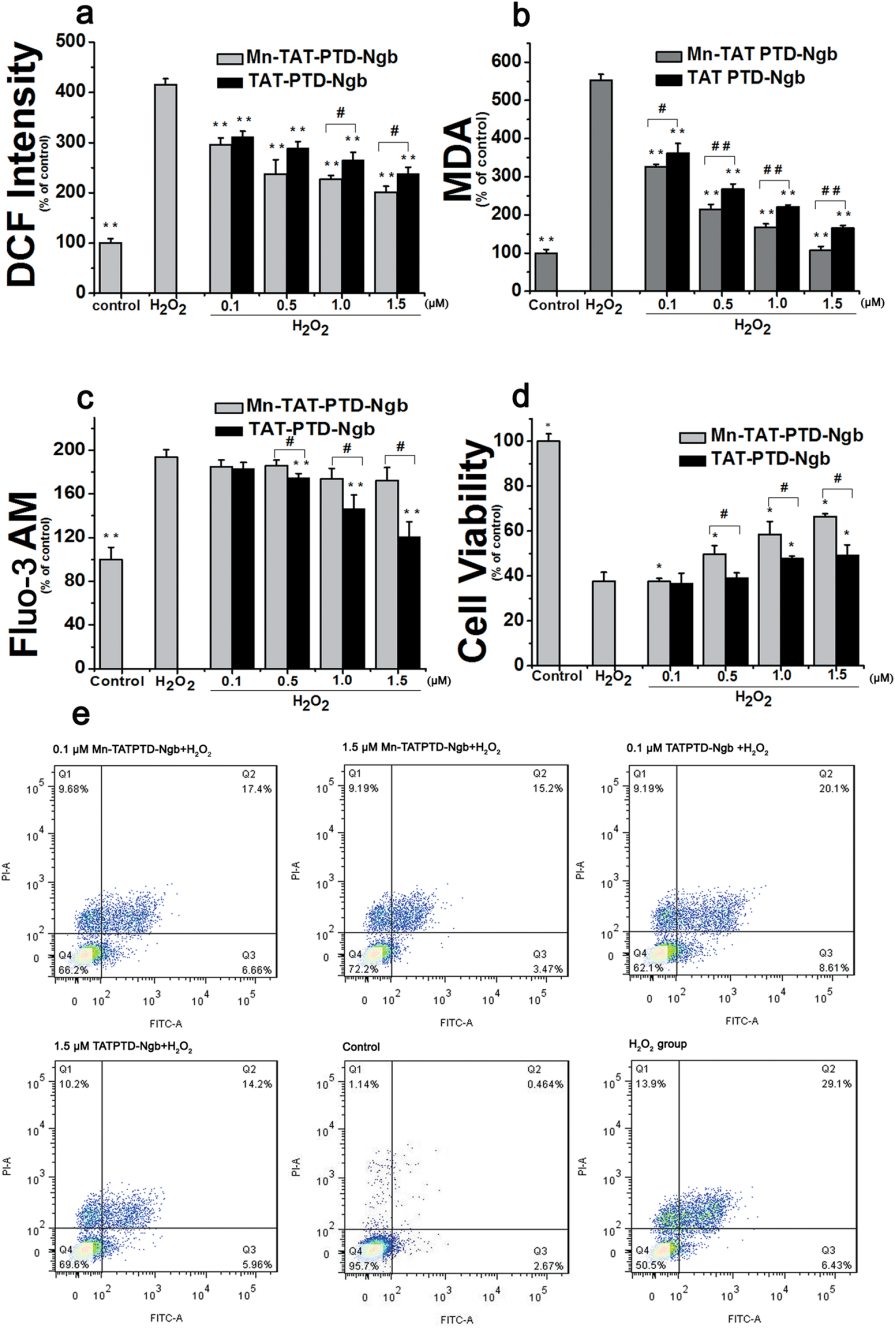
Mn-TAT PTD-Ngb attenuated H_2_O_2_-induced intracellular ROS levels. (**a)** Mn-TAT PTD-Ngb decreased the intracellular ROS levels of PC12 cells to protect against H_2_O_2_-induced injury and showed a better effect than that of TAT PTD-Ngb. PC12 cells were treated separately with Mn-TAT PTD-Ngb and TAT PTD-Ngb (0.1 μM, 0.5 μM, 1.0 μM, 1.5 μM). H_2_O_2_ was added to induce oxidative injury. PC12 cells were incubated with DCFH-DA, and the fluorescent intensity of DCF was measured by flow cytometry at an excitation wavelength of 488 nm. Data are expressed as the mean ± SD of three experiments (*p < 0.05 and **p < 0.01 compared to the H_2_O_2_ group, ^#^p < 0.05 and ^##^p < 0.01 compared to the TAT PTD-Ngb group). (**b**) Mn-TAT PTD-Ngb decreased H_2_O_2_-induced intracellular lipid peroxidation levels of PC12 cell. PC12 cells were treated with two fusion proteins and H_2_O_2_ as above. The cell lysates were collected and assayed according to commercial instruction. Data are expressed as the mean ± SD of three experiments (*p < 0.05 and **p < 0.01 compared to H_2_O_2_ group, ^#^p < 0.05 and ^##^p < 0.01 compared to TAT PTD-Ngb group). (**c**) Mn-TAT PTD-Ngb failed to improve H_2_O_2_-induce intracellular Ca^2+^ levels of PC12 cells. PC12 cells were treated with Mn-TAT PTD-Ngb and TAT PTD-Ngb (0.1 μM, 0.5 μM, 1.0 μM, 1.5 μM), respectively. After H_2_O_2_ treatment, cells were harvested and incubated with Fluo-3 AM, Mn-TAT PTD-Ngb showed no significant effect on relieving Ca^2+^stress. Data are expressed as the mean ± SD of three experiments. (*p < 0.05 and **p < 0.01 compared to H_2_O_2_ group, ^#^p < 0.05 and ^##^p< 0.01 compared to TAT PTD-Ngb group). (**d**) Mn-TAT PTD-Ngb elevated cell viability against H_2_O_2_-induced injury. PC12 cells were treated with two fusion proteins and H_2_O_2_, as above. Cell viability was determined by MTT assay. Data are expressed as the mean ± SD of three experiments (*p < 0.05 and **p < 0.01 compared to the H_2_O_2_ group, ^#^p< 0.05 and ^##^p< 0.01 compared to the TAT PTD-Ngb group). (**e**) Protective effect of Mn-TAT PTD-Ngb against H_2_O_2_-induced cell apoptosis (0.1 μM Mn-TAT PTD-Ngb + H_2_O_2_; 1.5 μM Mn-TAT PTD-Ngb + H_2_O_2_; 0.1 μM TAT PTD-Ngb + H_2_O_2_; 1.5 μM TAT PTD-Ngb + H_2_O_2_; Control; H_2_O_2_ group.).

DCFH-DA was used to detect intracellular ROS levels. DCFH in the cells was oxidized by ROS and the highly fluorescent DCF could be detected by flow cytometry (BD LSRFortessa). In H_2_O_2_ group, DCF fluorescent increased significantly compared with the control group (Fig. 3a). As Mn-TAT PTD-Ngb increased from 0.1 μM to 1.5 μM, the DCF intensity decreased gradually compared to the H_2_O_2_ group. The DCF intensity also decreased significantly in TAT-PTD-Ngb group.

#### Mn-TAT PTD-Ngb inhibits H_2_O_2_-induced lipid peroxidation

Lipid peroxidation is regarded as a biological marker of oxidative stress. Intracellular lipid peroxidation level increased greatly in H_2_O_2_ group compared with the control group (Fig. 3b). In the Mn-TAT PTD-Ngb group, the Malondialdehyde (MDA) level was reduced significantly, showing an inhibitory effect on intracellular lipid peroxidation levels and following a dose-dependent manner. TAT PTD-Ngb exhibited a less protective effect against H_2_O_2_ injury.

### Mn-TAT PTD-Ngb is not involved in relieving ER stress in H_2_O_2_-induced injury

Excessive ROS is closely associated with ER stress and disturbed intracellular Ca^2+^ homeostasis. Ca^2+^ level increased in the H_2_O_2_ group (Fig. 3c). The Ca^2+^ levels were almost invariable in the Mn-TAT PTD-Ngb group. But, in the TAT PTD-Ngb group, the Ca^2+^ levels decreased obviously, showing a better protective effect than the Mn-TAT PTD-Ngb group.

### Mn-TAT PTD-Ngb inhibits H_2_O_2_-induced apoptosis in PC12 cells

For the thiazolyl blue tetrazolium bromide (MTT) assay, PC12 cells were incubated with Mn-TAT PTD-Ngb or TAT-PTD-Ngb for 6 h, 300 μM H_2_O_2_ was added to induce oxidative injury. In Mn-TAT PTD-Ngb group, cell viability increased obviously and followed a dose-dependent manner (Fig. 3d). In TAT PTD-Ngb group, the cell viability increased little by little. This was also verified by the measurement of H_2_O_2_-induced cell apoptosis using Annexin V/PI double staining. In the Mn-TAT PTD-Ngb group, the apoptotic rate decreased from 27.20% to 19.16%, while the apoptotic rate of cells in the TAT PTD-Ngb group decreased from 28.54% to 21.32% (Fig. 3e).

### Mn-TAT PTD-Ngb improves mitochondrial function in H_2_O_2_-induced injury

#### Mn-TAT PTD-Ngb maintains the mitochondrial membrane potential

**Figure 4:**
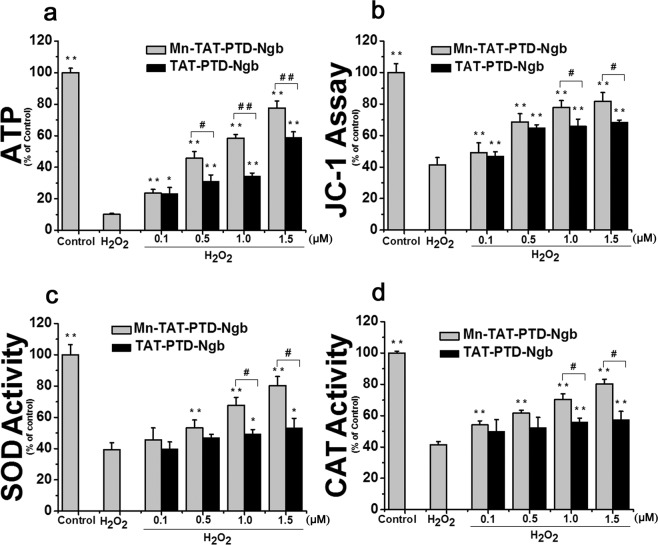
Mn-TAT PTD-Ngb maintained mitochondrial function and up-regulated intracellular SOD and catalase activity against H_2_O_2_-induced injury. (**a)** Mn-TAT PTD-Ngb maintained the intracellular mitochondrial membrane potential of PC12 cells against H_2_O_2_-induced injury and showed a better effect than that of TAT PTD-Ngb. PC12 cells were treated separately with Mn-TAT PTD-Ngb and TAT PTD-Ngb (0.1 μM, 0.5 μM, 1.0 μM, 1.5 μM). The fluorescent probe JC-1 was used to detect the monomer and aggregation on the surface of mitochondria. After H_2_O_2_ treatment, cells were harvested and incubated with JC-1. The ratio of the signal at 590 nm over 530 nm (red/green ratio) was calculated. Data are expressed as the mean ± SD of three experiments (* p < 0.05 and **p < 0.01 compared to the H_2_O_2_ group, ^#^p < 0.05 and ^##^p< 0.01 compared to the TAT PTD-Ngb group). (**b**) Mn-TATPTD-Ngb maintained the intracellular ATP levels in PC12 cells against H_2_O_2_-induced injury and showed a better effect than that of TAT PTD-Ngb. PC12 cells were treated with the two fusion proteins and H_2_O_2_ as above. The cell lysates were collected and assayed according to commercial instruction. Data are expressed as the mean ± SD of three experiments (*p < 0.05 and **p < 0.01 compared to the H_2_O_2_ group, ^#^p < 0.05 and ^##^p< 0.01 compared to the TATPTD-Ngb group). (**c, d)** Mn-TAT PTD-Ngb elevated the intracelluar SOD and CAT activities upon H_2_O_2_-induced injury of PC12 cells. PC12 cells were treated with the two fusion proteins and H_2_O_2_ as above. The cell lysates were collected and assayed according to the manufacturer’s instruction. Data are expressed as the mean ± SD of three experiments (*p < 0.05 and **p < 0.01 compared to the H_2_O_2_ group, ^#^p < 0.05 and ^##^p< 0.01 compared to the TAT PTD-Ngb group).

The mitochondrial membrane potential was detected using JC-1. The mitochondrial membrane potential was substantially down-regulated in the H_2_O_2_ group (Fig. 4a). Nevertheless, Mn-TAT PTD-Ngb and TAT PTD-Ngb could significantly improve the mitochondrial membrane potential, respectively.

#### Mn-TAT PTD-Ngb increases ATP levels

The ATP levels of cells reflect the degree of oxidative injury to the mitochondria. H_2_O_2_ decreased ATP levels (Fig. 4b). On the contrary, the ATP levels rose significantly after the treatment with Mn-TAT PTD-Ngb. Despite TAT PTD-Ngb could increase the ATP level by degrees, the protection effect was not as dramatic as that of Mn-TAT PTD-Ngb.

#### Mn-TAT PTD-Ngb decreases the mitochondrial ROS

Mitochondria were the main intracellular organelles that produced excessive ROS. Prolonged treatment of H_2_O_2_ induced the production of mitochondrial ROS (Fig. S4). The mitochondrial ROS levels were reduced under the treatment of Mn-TAT PTD-Ngb and followed a dose-dependent manner. Mn-TAT PTD-Ngb attenuated the oxidative injury to mitochondria and showed a more significantly effect on reducing mitochondrial ROS than that of TAT PTD-Ngb.

### Mn-TAT PTD-Ngb increases SOD and CAT enzyme activity in H_2_O_2_-induced injury

The antioxidant systems, including SOD and CAT, help cells to fight against excessive ROS injury. H_2_O_2_ can destroy the SOD activity. After the treatment with Mn-TAT PTD-Ngb, the SOD and CAT activities increased dramatically (Fig. 4c, d). However, TAT PTD-Ngb did not have a significant impact on the SOD and CAT activities.

### Mn-TAT PTD-Ngb attenuates H_2_O_2_-induced injury by inhibiting the mitochondrial apoptosis pathway and the activation of PI3K/Akt signaling pathway

**Figure 5:**
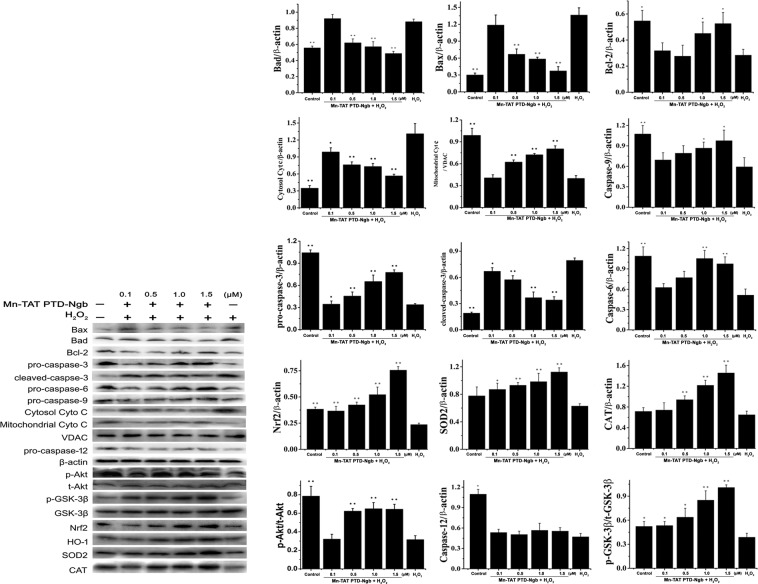
Mn-TAT PTD-Ngb participated in the mitochondrial and related signaling pathways to promote cell survival against H_2_O_2_-induced injury. (**a, b)** Mn-TAT PTD-Ngb reduced the protein levels of Bax, Bad, and Cyt c in cytosolic fraction; inhibited the cleavage of Casp-3, Casp-6, and Casp-9; and promoted the phosphorylation of Akt and GSK-3β in the PI3K/Akt pathway and up-regulated the levels of Nrf2, HO-1, SOD2, CAT, but had no significant effect on inhibiting Csp-12 cleavage. PC12 cells were incubated with Mn-TAT PTD-Ngb for 6 h after H_2_O_2_ treatment, and the cell lysates were analyzed by Western blot. The amount of protein was normalized with β-actin levels. Data are expressed as the mean ± SD of three experiments (*p < 0.05 and **p < 0.01 compared to the H_2_O_2_ group). The exposure time of each blot was 10 s.

**Figure 6:**
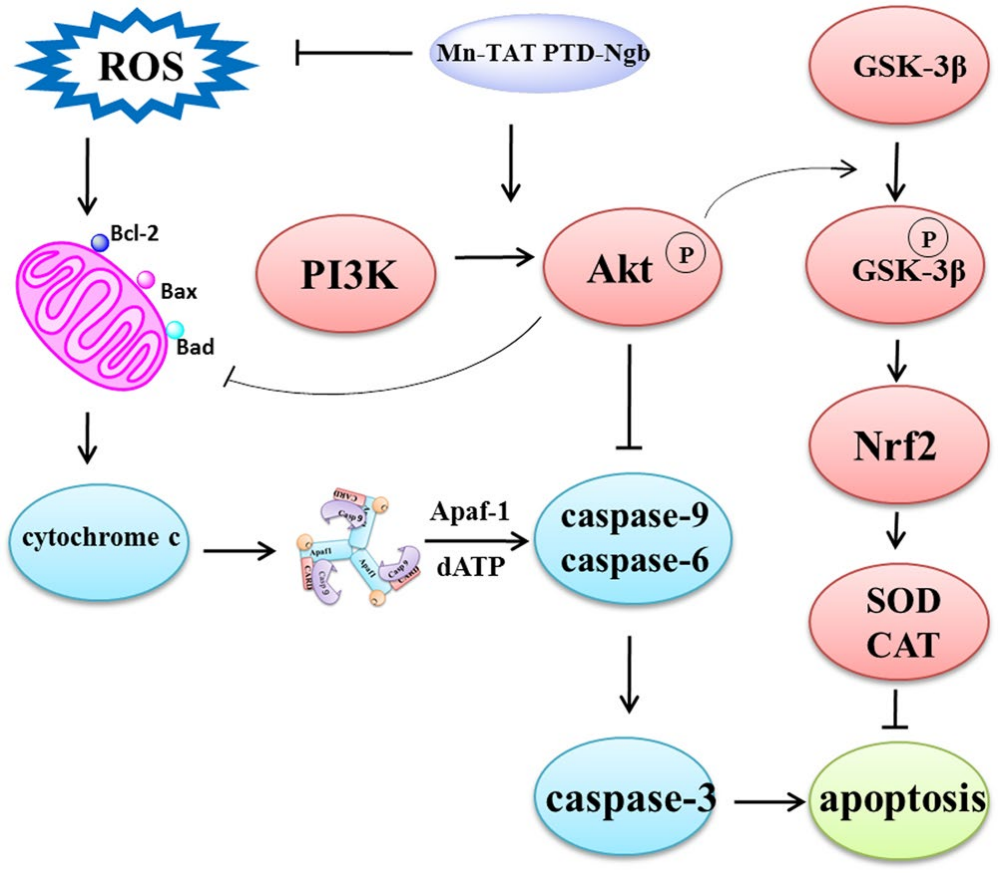
Schematic illustration of the presumed mechanism of the protective effect of Mn-TAT PTD-Ngb against oxidative injury in PC12 cells.

To illuminate the neuroprotective effect of Mn-TAT PTD-Ngb, we analyzed the expression of key apoptotic factors related to the mitochondrial signaling pathway and PI3K/Akt signaling pathway by Western blot (Fig. 5). Mn-TAT PTD-Ngb upregulated the anti-apoptotic factor Bcl-2 and downregulated pro-apoptotic factors, such as Bad and Bax. Mn-TAT PTD-Ngb inhibited the release of Cyt c from mitochondria into cytosol and activation of caspases 3, 6 and 9^37^. In PI3K/Akt signaling pathway, Mn-TAT PTD-Ngb promoted the phosphorylation of Akt and GSK-3β, which was also verified by perifosine (Figs. S5 and S6). Mn-TAT PTD-Ngb could attenuate the phosphorylation of Akt and its downstream signaling molecule (GSK-3β) inhibited by the perifosine. Therefore, it led to increase the expression of downstream Nrf2, HO-1, SOD, CAT (Figs. 5 and S5). This favored the eliminating of ROS and the promoting of cell survival. These results suggested that Mn-TAT PTD-Ngb inhibited mitochondrial-mediated apoptosis and promoted cell survival by PI3K/Akt signaling pathway (Fig. 6). However, Mn-TAT PTD-Ngb did not obviously regulate the activation of caspase-12, which participated in ER stress-induced apoptosis.

## Discussion

Neurological diseases, such as stroke, traumatic brain injuries and Alzheimer, are closely related to the interruption of redox homeostasis. Ngb, a recently identified globin, is reported to get involved in redox biology and plays important role in protecting against neurological diseases. The heme in this globin was replaced by manganese porphyrin, bringing with a more negative potential than TAT PTD-Ngb. Mn-TAT PTD-Ngb showed stronger ability for reducing ROS, and modulated the mitochondrial function and redox balance in controlling of apoptotic death. Mn-TAT PTD-Ngb was presumed to be one of the redox signaling molecules that prevented the activation of mitochondria-dependent apoptosis and facilitated the activation of PI3K/Akt to promote cell survival.

Spectroscopic analysis of Mn-TAT PTD-Ngb and TAT PTD-Ngb indicated the reconstitution of the metal porphyrin and reconstitution of the metal porphyrin and scaffold mainly retained the secondary structure of TAT PTD-Ngb (Fig. 2b and Table 1). By measuring cyclic voltammetry to study the Mn-TAT PTD-Ngb electrochemical properties, Mn-TAT PTD-Ngb revealed a relatively more negative potential than TAT PTD-Ngb (Fig. 2e, f), which provided the redox chemistry of antioxidant effects for Mn-TAT PTD-Ngb. Ngb acted as an anti-oxidative enzyme in protecting neurons^38^. In SH-SY5Y cells up-regulating Ngb levels attenuated H_2_O_2_-induced injury^39^ and reduced ROS production and lipids peroxidation in beta-amyloid-induced injury in PC12 cells^40^. There were reduced infarcts size and cell apoptosis in focal cerebral ischemia with up-regulated Ngb levels^41^. As Ngb exhibited antioxidant ability *in vitro* and *in vivo*^14,42^, the ROS scavenging ability of Mn-TAT PTD-Ngb in PC12 cells was determined. H_2_O_2_could increase the accumulation of ROS and the production of lipid peroxidation. Mn-TAT PTD-Ngb ameliorated the ROS and lipid peroxidation levels, exhibiting a better effect than that observed with TAT PTD-Ngb (Fig. 3a, b). Mitochondrion was the center for redox signals. ROS signals were amplified by collapsed mitochondrial membrane potential, loss of ATP, release of mitochondrial ROS and pro-apoptosis proteins (Figs. 4a, b and 5). The results showed Mn-TAT PTD-Ngb significantly reduced mitochondrial ROS, showing a better effect than TAT PTD-Ngb (Fig. S4). Given that pro-apoptosis proteins Bax and Bad permeability were located on the mitochondrial membrane and deteriorated mitochondrial membrane permeability, anti-apoptotic protein Bcl-2 and pro-apoptosis proteins Bad and Bax levels were measured. The data showed Mn-TAT PTD-Ngb down-regulated of the pro-apoptosis proteins Bax and Bad and up-regulated Bcl-2, which prevented H_2_O_2_–induced mitochondrial membrane damage and ATP loss. It was observed that ATP loss and collapsed mitochondrial membrane potential were attenuated by Mn-TAT PTD-Ngb. Mn-TAT PTD-Ngb treatment favored the restoring of impaired mitochondrial membrane permeability. Persistent ROS production led to apoptosis, the effect of Mn-TAT PTD-Ngb on H_2_O_2_ -induced cell apoptosis was studied. The release of Cyt c from mitochondria to cytosol upon oxidative injury was inhibited by Mn-TAT PTD-Ngb and followed a dose-dependent manner (Fig. 5). The pro-caspase-9 and pro-caspase-6 enzymes were downstream of Cyt c, and their cleavages were inhibited by Mn-TAT PTD-Ngb, leading to the inhibition of the apoptosis executer caspase-3. It suggested that activation of mitochondria-dependent apoptotic pathway was suppressed by Mn-TAT PTD-Ngb, which was similar to that of Ngb.

PI3K/Akt was an important signaling pathway to protect neurons against ischemia-reperfusion injury in the brain. Phosphorylation of Akt participated in anti-apoptotic signaling by inhibiting Bax, pro-caspase-9 cleavage and up-regulating Bcl-2. There was increased expression of phosphorylation of Akt in Mn-TAT PTD-Ngb group. It was observed that the downstream Bax, Bad and pro-caspase-9 cleavage were suppressed under Mn-TAT PTD-Ngb treatment and Bcl-2 levels were up-regulated, following a dose-dependent manner. GSK-3β was a negative regulator of Nrf2, phosphorylation of GSK-3β led to up-regulated Nrf2, which played critical roles in regulating redox signaling and attenuating ischemia injury^43^. It was observed that Mn-TAT PTD-Ngb increased the phosphorylation of GSK-3β, which led to up-regulated Nrf2, HO-1 expression and the up-regulation of downstream SOD, CAT (Figs. 5 and S5). This enhanced the effectiveness of attenuating oxidative injury and was verified by the decline of ROS and lipid peroxidation production (Fig. 3a,b), release of mitochondrial ROS, and the elevated expression and activities of SOD and CAT (Figs. S4 and 4c, d).

In conclusion, the activation of PI3K/Akt and inhibition of mitochondrial apoptotic pathway played important roles in protecting cells against oxidative injury, leading to increase cell viability and inhibit apoptosis (Fig. 3d, e).

However, Mn-TAT PTD-Ngb showed no significant effect on relieving ER stress induced by oxidative injury. Caspase-12 is located on the ER and participates in ER stress-induced apoptosis. The cleavage of pro-caspase-12 was up-regulated in the H_2_O_2_ group and Mn-TAT PTD-Ngb did not have a significant effect in reducing the cleavage of caspase-12 (Fig. 5). Additionally, H_2_O_2_ treatment increased the release of Ca^2+^. Mn-TAT PTD-Ngb failed to inhibit the release of Ca^2+^, while TAT PTD-Ngb showed a significant protective effect (Fig. 3c). This implied that Mn-TAT PTD-Ngb did not inhibit cell apoptosis by relieving the ER stress, while TAT PTD-Ngb did.

Due to the manganese redox chemistry in biological systems, it eliminates ROS as the superoxide dismutase, bacteria catalases in biological systems^25,44,45^. Like most Mn-porphyrin and its derivatives, Mn-TAT PTD-Ngb was presumed to be a highly efficient scavenger of intracellular ROS. It reduced intracellular ROS levels, mitochondrial ROS release and down-regulated pro-apoptosis molecule that impaired mitochondrial membrane permeability. Besides, Mn-TAT PTD-Ngb activated PI3K/Akt signaling pathway and up-regulated Nrf2, HO-1, SOD, CAT, which significantly attenuated intracellular ROS production (Fig. 6). It clearly showed that Mn-TAT PTD-Ngb regulated redox signaling and this was not merely relied on its redox chemistry in biological systems. There were redox regulations by multiple signaling pathways such as PI3K/Akt with enhanced SOD, CAT activity. Mn-TAT PTD-Ngb displayed enhanced antioxidant abilities by the replace of redox center of manganese. Still, Mn-TAT PTD-Ngb retained similar signaling pathway, it might be attributed to that Ngb scaffold contains many binding sites for multiple intracellular receptors. However, the changes of metal center also brought with some changes like the loss of relieving ER stress.

Currently, more study of exploiting the therapy of neurological diseases is in urgent need. The redox regulation of metal center of protein may provide more information on the design and synthesis of new drugs for the therapeutic treatment of neurological diseases.

## Materials and Methods

### Regents

*E.coli *BL21(DE3)plysS was bought from Novagen company. Mn(III)-protoporphyrin IX was obtained from Frontier Scientific, Inc(USA). PC12 cells were from the Cell Bank of the Chinese Academy of Sciences. Isopropyl β-D-1-thiogalactopyranoside (IPTG), phenylmethylsulfonyl fluoride (PMSF) and kanamycin were bought from Biosharp (China). Fetal bovine serum (FBS) was bought from HyClone (Australia), Dulbecco’s Modified Eagle’s medium (DMEM) and the double staining apoptosis kit were bought from KeyGEN (China). MTT was purchased from Sigma (USA). Dimethyl sulfoxide (DMSO) was purchased from ANPEL Laboratory Technologies Inc (China). Perifosine was purchased from Meilunbio (China). ATP assay kit, catalase assay kit, total superoxide dismutase assay kit with WST-8, radio immunoprecipitation assay (RIPA) lysis buffer, mitochondrial membrane potential assay kit with JC-1, Fluo-3 AM kit, oxygen species assay kit, lipid peroxidation MDA assay kit, cell mitochondria isolation kit, Hoechst 33342 were purchased from Beyotime Institute of Biotechnology (China). MitoSOX Red mitochondrial superoxide indicator was obtained from Invitrogen (USA). Antibodies against β-actin, caspase-3, caspase-9, caspase-12, caspase-6, Bax, Bad, Bcl-2, Cyt c, GSK-3β, p-GSK-3β, SOD were bought from Cell Signaling Technology (USA), total-Akt (t-Akt), phospho-Akt (p-Akt) were bought from ImmunoWay (USA), Nrf2 was bought from Abcam (UK), CAT, HO-1 were bought from Absin (China). Anti-6×His-tag was bought from Sangon Biotech (China). Secondary antibody anti-mouse IgG, anti-rabbit IgG were bought from Cell Signaling Technology (USA), Chemiluminescent HRP substrate was bought from Millipore (USA). The gold electrode was bought from Chen hua (China). All reagents were of analytical grade and all solutions were prepared using Milli-Q deionized water.

### Construction of the TAT PTD-Ngb plasmid and expression of the fusion protein

An Ngb gene corresponding to the sequence of the cDNA clone, which contains the TAT PTD sequence, was chemically synthesized with 5′ NcoI and 3′ BamHI sites as well as codon optimization for expression in *E. coli* and was subcloned into the EcoRV site of pUC57-Kan (GenScript, China). The synthetic Ngb gene was cloned as an NcoI/BamHI digest from pUC57-Kan-TAT PTD-Ngb into the expression vector pET30a (+) to produce a fusion with six histidine residues at the 5′ end. The expression plasmid was transformed into *E. coli* BL21 (DE3)plysS. The *E. coli* BL21 (DE3) cells were cultured in terrific broth (TB) medium with kanamycin (0.1 mg/ml) at 37 °C. When the bacterial culture medium OD reached 0.8, IPTG (0.1 mg/ml) was added and bacteria were cultivated at 20 °C and 120 rpm for 12 h.

### Purification of the TAT PTD-Ngb fusion protein

Bacteria were harvested by centrifugation at 5000 rpm and 4 °C for 15 min and washed with Milli-Q deionized water. The bacteria were solubilized in 1× binding buffer with lysozyme and lysed in an ultrasonic cell disruptor in an ice-bath. Homogenates were centrifuged at 4 °C and 10000 rpm for 30 min, and the precipitates were discarded. The fusion protein in the supernatants was purified with Ni-NTA resin and desalted with a G-25 column. The supernatant, precipitate and purified proteins were analyzed by SDS-PAGE.

### Reconstitution of apo-TAT PTD-Ngb with Mn(III)-protoporphyrin IX

Apo-TAT PTD-Ngb was prepared from TAT PTD-Ngb using the acid-methylethylketone method described in previous reports. The reconstitution of apo-TAT PTD-Ngb with Mn(III)-protoporphyrin IX was according to previous reports. Mn(III)-protoporphyrin IX was dissolved in 100 mM NaOH. A 2 molar equivalent amount of Mn(III)-protoporphyrin IX was added into an apo-TAT PTD-Ngb phosphate buffer solution (in 50 mM pH 7.4), and the solution was kept at 4 °C for 12 h. A Sephadex G-25 column was pre-equilibrated with a 50 mM phosphate buffer solution (pH 7.4). The mixture was loaded onto a Sephadex G-25 column to remove excessive Mn(III)-protoporphyrin IX. The concentration of Mn-TAT PTD-Ngb was determined by UV spectroscopy at a wavelength of 280 nm.

### Western-blot analysis

PC12 cells were seeded in 60 mm dishes at the density of 1 × 10^5^ cells/ml. The cells were incubated with two fusion proteins for 6 h and subsequently H_2_O_2_ (300 μM) for 5 h, respectively. The cells were washed with cold PBS for three times and lysed by RIPA lysis buffer with PMSF and cocktail under ice-bath. Protein concentrations were determined by BCA protein assay kit (Beyotime Institute of Biotechnology). Equal amounts of proteins were analyzed in 12% or 15% polyacrylamide gels by SDS-PAGE and subsequently transferred to PVDF membranes. PVDF membranes were blocked with 5% nonfat milk in PBST (PBS with 0.1% Tween 20, pH 7.4) and incubated with primary antibody overnight at 4 °C (caspase-3, caspase-9, caspase-6, caspase-12, Bcl-2, β-actin, Bad, Bax, Cyt c, Anti-6 × His-tag, GSK-3β, p-GSK-3β, SOD2, t-Akt, p-Akt, Nrf2, CAT, HO-1, 1:1000). Subsequently, PVDF membranes washed with PBST and incubated with secondary antibody for 1 h at room temperature. Signals were analyzed by Chemiluminescence (UVP BioSpectrum) with horseradish peroxidase-conjugated IgG (Millipore).

### Measurement of intracellular ROS production

The intracellular ROS levels were measured by DCF that the oxidation production of fluorescent probe DCFH-DA (Beyotime Institute of Biotechnology). The fluorescence was measured by flow cytometer (BD LSRFortessa) at the excitation and emission wavelengths of 485 nm and 528 nm, respectively. PC12 cells were treated with TAT PTD-Ngb, Mn-TAT PTD-Ngb and H_2_O_2_ as above experiments. Then cells were collected and incubated with DCFH-DA at 37 °C for 20 min. The cells were washed with PBS for 3 times to remove excessive DCFH-DA. The fluorescence of DCF was recorded by flow cytometer (BD LSRFortessa). The data were expressed as a percentage of the fluorescence of the control group.

### Catalase activity analysis

Catalase activity was analyzed by catalase assay kit (Beyotime Institute of Biotechnology). PC12 cells were seeded in six well plates. After the same treatment of two fusion proteins and H_2_O_2_ as above experiments, the cells were washed with cold PBS and lysed with RIPA lysis buffer (Beyotime Institute of Biotechnology). The cell lysates were centrifuged at 1, 600 rpm 4 °C for 20 min, the supernatant was diluted to proper concentration and protein concentrations were determined by BCA protein assay kit (Beyotime Institute of Biotechnology). The catalase activity was measured following the kits instruction by an automatic enzyme-linked immunosorbent assay plate reader (Thermo Scientific Varioskan Flash) at the wavelength of 520 nm. These data were expressed in percentage change compared with the control.

### SOD activity analysis

SOD activity was measured by the total superoxide dismutase assay kit with WST-8 (Beyotime Institute of Biotechnology). PC12 cells were seeded in six well plates. After the same treatment of two fusion proteins and H_2_O_2_ as above experiments, the cells were washed with cold PBS and harvested. The cells were suspended with cold PBS and lysed by the ultrasonic cell disruptor. Protein concentrations were determined by BCA protein assay kit (Beyotime Institute of Biotechnology). The SOD activity was measured following the kits instruction by an automatic enzyme-linked immunosorbent assay plate reader (Thermo Scientific Varioskan Flash) at the wavelength of 450 nm. These data were expressed in percentage change compared with the control.

### Mitochondrial membrane potential

The mitochondrial membrane potential was detected by JC-1 fluorescent dye kit (Beyotime Institute of Biotechnology). PC12 cells were treated with two fusion proteins and H_2_O_2_ as above experiments. The cells were collected and washed with cold PBS. Then the cells were suspended in DMEM and incubated with JC-1 for 20 min at 37 °C. Cells were washed with JC-1 dying buffer for twice and suspended in JC-1 dying buffer. The mitochondrial membrane potential was measured by flow cytometer (BD LSRFortessa) at the excitation of 488 nm and at the emission wavelength of 530 nm and 590 nm. The ratio of emission wavelengths at 590 nm and 530 nm (red/green ratio) was calculated. The data were expressed as ratio of red to green fluorescence.

### Intracellular ATP assay

The intracellular ATP levels were analyzed by ATP assay kit (Beyotime Institute of Biotechnology). The cells were seeded in 6-well plates and treated with two fusion proteins and H_2_O_2_ as above experiments. The cells were washed with cold PBS and lysed with RIPA lysis buffer (Beyotime Institute of Biotechnology). Cell lysates were centrifuged at 12, 000 g 4 °C for 10 min, the supernatant was used to analyze the ATP levels by the commercial kits instruction. Protein concentration was determined by BCA protein assay kit (Beyotime Institute of Biotechnology). Luminance was measured by an automatic enzyme-linked immunosorbent assay plate reader (Thermo Scientific Varioskan Flash). These data were expressed in percentage change compared with the control.

### Separation of mitochondria

The mitochondria were isolated by cell mitochondria isolation kit (Beyotime Institute of Biotechnology). PC12 cells were seeded in 60 mm dishes at the density of 1 × 10^5^ cells/ml. The cells were treated with two fusion proteins and H_2_O_2_ as above experiments. PC12 cells were washed with cold PBS for three times and homogenized with mitochondria extraction reagent supplemented with PMSF under ice-bath. The homogenates were centrifuged at 600 g for 10 min, and then the supernatants were further centrifuged at 11, 000 g for 10 min. The resulting supernatants contained cytosolic fractions were collected. The precipitates contained mitochondrial fractions were lysed with mitochondrial lysis buffer. Protein concentrations were determined by BCA protein assay kit (Beyotime Institute of Biotechnology). The cytosolic and mitochondrial Cyt c levels in PC12 cells were analyzed by Western blot.

### Cell viability assay

Cell viability was determined by MTT assay. PC12 cells were cultured in 96-well plates and treated with two fusion proteins and H_2_O_2_ as above experiments. 0.5 mg/ml MTT was added and incubated at 37 °C for 4 h. After removing the medium, 150 μl DMSO was added to dissolve formazan crystals. The viability rate of PC12 was measured by an automatic enzyme-linked immunosorbent assay plate reader (Thermo Scientific Varioskan Flash) at the wavelength of 570 nm. These data were expressed a percentage of the control.

### Statistical analysis

Data are expressed as mean ± SD. Student’s T. Test was used to compare two independent groups. A probability of P < 0.05 was considered statistically significant.

## Supplementary information

supplementary materials

## Data Availability

Data have been submitted as Supplementary Materials.
