# Supplementary material for: Mn-TAT PTD-Ngb attenuates oxidative injury by an enhanced ROS scavenging ability and the regulation of redox signaling pathway
Source: Sci Rep. 2019 Dec 27;9:20103. doi: 10.1038/s41598-019-56595-5 (PMC6934865; doi:10.1038/s41598-019-56595-5)
Supplement: Supplementary file 1 — supplementary materials [file 41598_2019_56595_MOESM1_ESM.pdf]

Online Supplement

Mn-TAT PTD-Ngb attenuates oxidative injury by an enhanced ROS scavenging ability and the regulation of redox signaling pathway

Cui Zhang, Xuehui Hao, Jiaying Chang, Zhirong Geng*, Zhilin Wang*

State key Laboratory of Coordination Chemistry, School of Chemistry and Chemical Engineering, Collaborative Innovation Center of Advanced Microstructures, Nanjing University, Nanjing 210023, P.R. China.

**Corresponding authors: Zhirong Geng and Zhilin Wang, State key Laboratory of Coordination Chemistry, School of Chemistry and Chemical Engineering, Collaborative Innovation Center of Advanced Microstructures, Nanjing University, Nanjing 210023, P.R. China. Tel.: +86-25-89687761; Fax: +86-25-89689007. E-mail: gengzr@nju.edu.cn, wangzl@nju.edu.cn*

1. Materials and Methods

Cell culture

PC12 cells were cultured in DMEM medium, seeded at a density of 1×10^6 cells/ml in 100 mm poly-L-lysine-coated plastic culture dishes and cultivated at 37 °C in a 5% CO_2 atmosphere. The

medium was removed, and fresh medium was added every day. The cells were maintained for 3 days before the experiments.

CV (Cyclic voltammetry) measurement

Electrochemical experiments were measured by CHI600D (Chenhua Instruments Co., China). The electrochemical system was a conventional three-electrode system including: the working gold electrode, counter platinum wire-coil and the reference electrode Ag|AgCl|3M KCl. The gold electrode was polished by 0.075 μM alumina and washed with Milli-Q deionized water for three times. The gold electrode was modified with 10 mM cysteine for 8 h and mercaptoethanol for 12 h. The modified electrode was washed with Milli-Q deionized water and immersed in 30 mM TAT PTD-Ngb and Mn-TAT PTD-Ngb solution respectively at 4 °C for 12 h. The cyclic voltammetric experiments were measured under the anaerobic N_2 protection, the scan rate was 100 $mV \cdot s^{-1}$.

Translocation of Mn-TAT PTD-Ngb into PC12 cells

PC12 cells were seeded in 60 mm dishes at the density of 1×10^5 cells/ml. The cells were incubated with Mn-TAT PTD-Ngb for 6 h at the concentration of 0.1 μM, 0.5 μM, 1.0 μM, 1.5 μM, respectively. The cells were washed with PBS containing 0.2% Triton X-100 for three times to remove excessive Mn-TAT PTD-Ngb, and then washed with cold PBS for three times. PC12 cells were lysed by RIPA lysis buffer with protease inhibitors (PMSF) and cocktail under ice-bath. The intracellular Mn-TAT PTD-Ngb levels of PC12 cells were detected by Western blot.

To investigate the effect of incubation time on the translocation of Mn-TAT PTD-Ngb into PC12 cells, 1.0 μM Mn-TAT PTD-Ngb was incubated with Mn-TAT PTD-Ngb for 2 h, 6 h, 24 h, 36 h, respectively. PC12 cells were harvested and lysed as above experiments. The intracellular Mn-TAT PTD-Ngb levels of PC12 cells were detected by Western blot.

Lipid peroxidation assay

Lipid peroxidation levels in PC12 cells were analyzed by lipid peroxidation MDA assay kit (Beyotime Institute of Biotechnology). PC12 cells were seeded in six well plates, incubated with TAT-PTD-Ngb and Mn-TAT PTD-Ngb (0.1μM, 0.5 μM, 1.0 μM, 1.5 μM) for 6 h. H_2O_2 (300 μM) was added and incubated for 5 h subsequently. The cells were washed with cold PBS and lysed with RIPA lysis buffer under ice-bath. The collected total cell lysates were centrifuged at 1600 rpm 4 °C for 20 min, and the supernatant was used to determine the lipid peroxidation levels of PC12 cells. Protein concentrations were determined by BCA protein assay kit (Beyotime Institute of Biotechnology). The MDA was measured following the commercial kits instruction. MDA levels were expressed as μM per mg of protein.

Determination of mitochondrial ROS levels

Mitochondrial superoxide levels were measured using MitoSOX Red (Invitrogen M36008). PC12 cells were seeded in glass-bottomed dishes and pretreated with two fusion proteins and H_2O_2 as above experiments. PC12 cells were incubated with 200 nM MitoSOX Red for 10 min at 37 °C and washed 3 times with PBS. Hoechst 33342 (5 μg/ml) was added to stain the nuclei for 20 min. Images were captured by a Zeiss 510 Meta confocal laser scanning microscope (LSM 710) (excitation 488 nm, emission 525 nm).

Intracellular Ca^{2+} assay

Fluo-3 AM was a fluorescent probe that could be used to detect the intracellular Ca^{2+} levels of cells. Ca^{2+} was measured by Fluo-3 AM kit (Beyotime Institute of Biotechnology). PC12 cells were

treated with two fusion proteins and H_2O_2 as above experiments. Cells were harvested and washed with cold PBS, Fluo-3 AM was added and incubated for 30 min at 37 °C. Then cells were washed twice with cold PBS and incubated for another 30 min at 37 °C to make sure that Fluo-3 AM could completely turn to Fluo-3. The fluorescent of Fluo-3 was measured at the excitation of 488 nm and the emission of 530 nm by flow cytometry (BD LSRFortessa). These data were expressed as a percentage of the fluorescence of the control group.

Cell apoptosis assay

For cells apoptotic analysis, the PC12 cells were seeded in a 6-well culture plate at a concentration of 1×10^4 cells/well and cultured in DMEM medium and 10% FBS. After treatment of two fusion proteins and H_2O_2 as above experiments, the cells were harvested and washed twice with ice-cold PBS, and then re-suspended in the dark with AnnexinV-FITC and PI (KeyGEN Biotech, China) buffer for 15 min at room temperature. Apoptosis cells were analyzed with a flow cytometry (BD LSRFortessa). Cells were considered to be apoptotic when they were either Annexin V+/PI− (early apo-ptotic) or Annexin V+/PI+ (late apoptotic).

The effects of Mn-TAT PTD-Ngb on the phosphorylation of Akt and its downstream signaling molecule GSK-3β in PC12 cells

Perifosine was an Akt phosphorylation inhibitor and was bought from Meilunbio (MB1880). PC12 cells were pretreated with perifosine (10 μM) for 30 min, then the cells were incubated with Mn-TAT PTD-Ngb for 6 h and subsequently with the treatment of H_2O_2 (300 μM) for 5 h. The cells were washed with cold PBS for three times and lysed by RIPA lysis buffer with protease inhibitors (PMSF) and cocktail under ice-bath. Protein concentrations were determined by BCA protein assay kit

(Beyotime Institute of Biotechnology). The effects of Mn-TAT PTD-Ngb on the phosphorylation of Akt and its downstream signaling molecule GSK-3β were analyzed by Western blot.

2. Supplemental Figures

ccATGGGTAGCAGCCATCATCATCATCATCATCTGGTTCCGCGCGGTAGCTATGGTCGGAAGAAACGTC
GCCAGCGTCGCCGCATGGAACGTCCGGAACCGGAACTGATTCGTCAGAGCTGGCGCGCAGTTAGCCGTA
GCCCGCTGGAACATGGCACTGTTCTGTTTGCACGCCTGTTTGCACTGGAACCGGATCTGCTGCCGCTGT
TTCAGTATAATTGTCGCCAGTTTAGCAGCCCGGAAGATTGTCTGAGCAGCCCGGAATTTCTGGATCATA
TTCGTAAAGTTATGCTGGTTATTGATGCAGCAGTTACCAATGTTGAGGACCTGAGCAGCCTGGAAGAAT
ATCTGGCAAGCCTGGGTCGCAAACATCGTGCAGTTGGTGTTAAACTGAGCAGCTTTAGCACCGTTGGTG
AAAGCCTGCTGTATATGCTGGAAAAATGTCTGGGTCCGGCCTTTACCCCGGCAACCCGTGCAGCATGGA
GCCAGCTGTATGGTGCAGTTGTTCAGGCAATGAGCCGCGGTTGGGATGGTGAATAAggatcc

Fig. S1. DNA sequence of TAT PTD-Ngb.

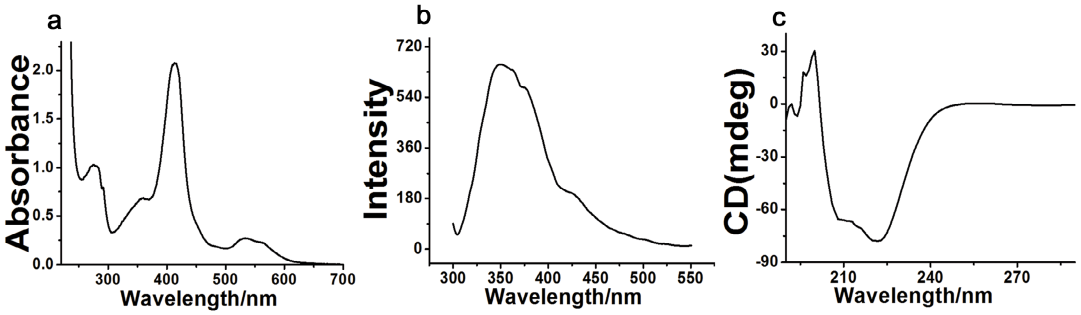

Fig. S2. Spectral analysis of TAT PTD-Ngb. **a** UV-Vis spectrum of TAT PTD-Ngb. **b** Fluorescence spectrum of TAT PTD-Ngb. **c** Circular dichroism spectrum of TAT PTD-Ngb.

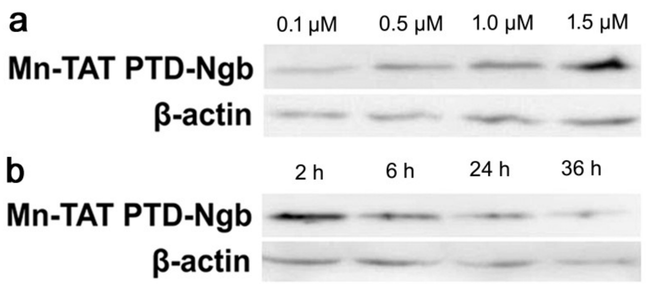

Fig. S3. Translocation of Mn-TAT PTD-Ngb into PC12 cells. **a** Mn-TAT PTD-Ngb delivered into PC12 cells in a dose-dependent manner. **b** Mn-TAT PTD-Ngb delivered into PC12 cells in a time-dependent manner. The samples were derived from the same experiment and the blots were processed in parallel. The exposure time of each blot was 10 s.

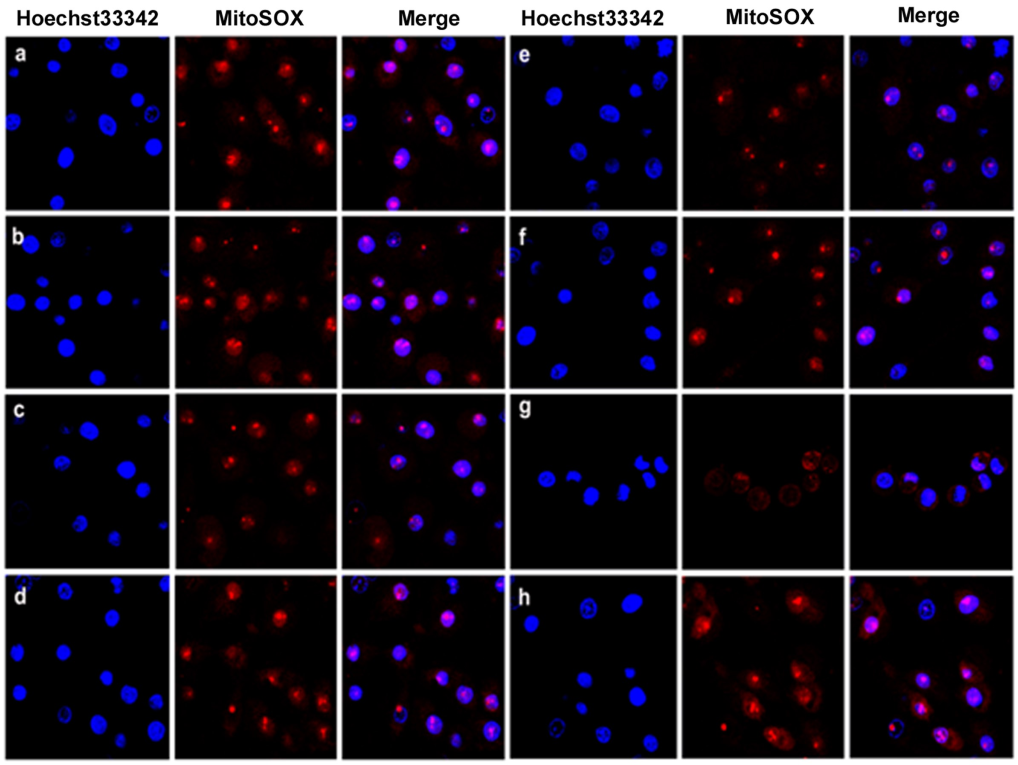

Fig. S4. Mn TAT PTD-Ngb inhibited H_2O_2-induced mitochondrial ROS levels. PC12 cells were treated with TAT PTD-Ngb and Mn TAT PTD-Ngb for 6 h, respectively. PC12 cells were stimulated with H_2O_2 (300μM), and then stained with MitoSOX Red for 10 min and Hoechst 33342 (5 μg/ml) for 20 min and analysed by a Zeiss 510 Meta confocal laser scanning microscope (LSM 710).

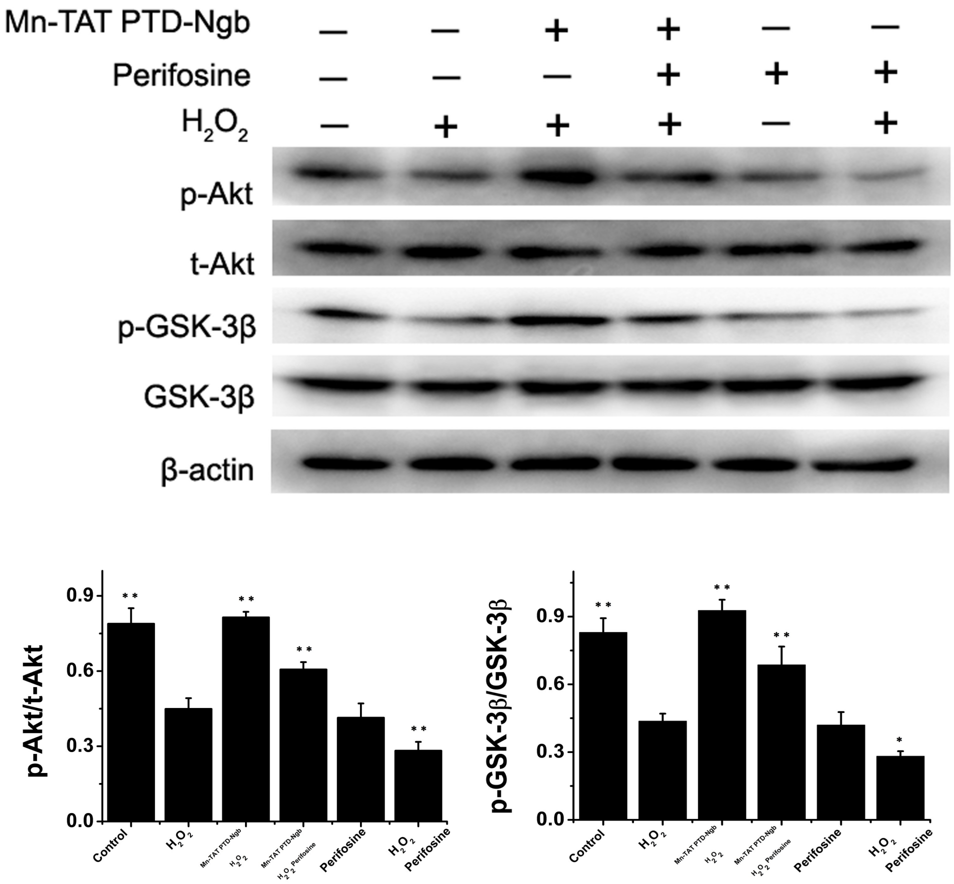

Fig. S5. Mn-TAT PTD-Ngb promoted the phosphorylation of Akt and its downstream signaling molecule GSK-3β in PC12 cells. PC12 cells were pretreated with perifosine (10 μM) for 30 min, then the cells were incubated with Mn-TAT PTD-Ngb for 6 h and subsequently with the treatment of H_2O_2 (300 μM) for 5 h. The effects of Mn-TAT PTD-Ngb on the phosphorylation of Akt and its downstream signaling molecule GSK-3β were analyzed by Western blot. (* $p < 0.05$ and ** $p < 0.01$ compared to the perifosine group). The exposure time of each blot was 10 s.

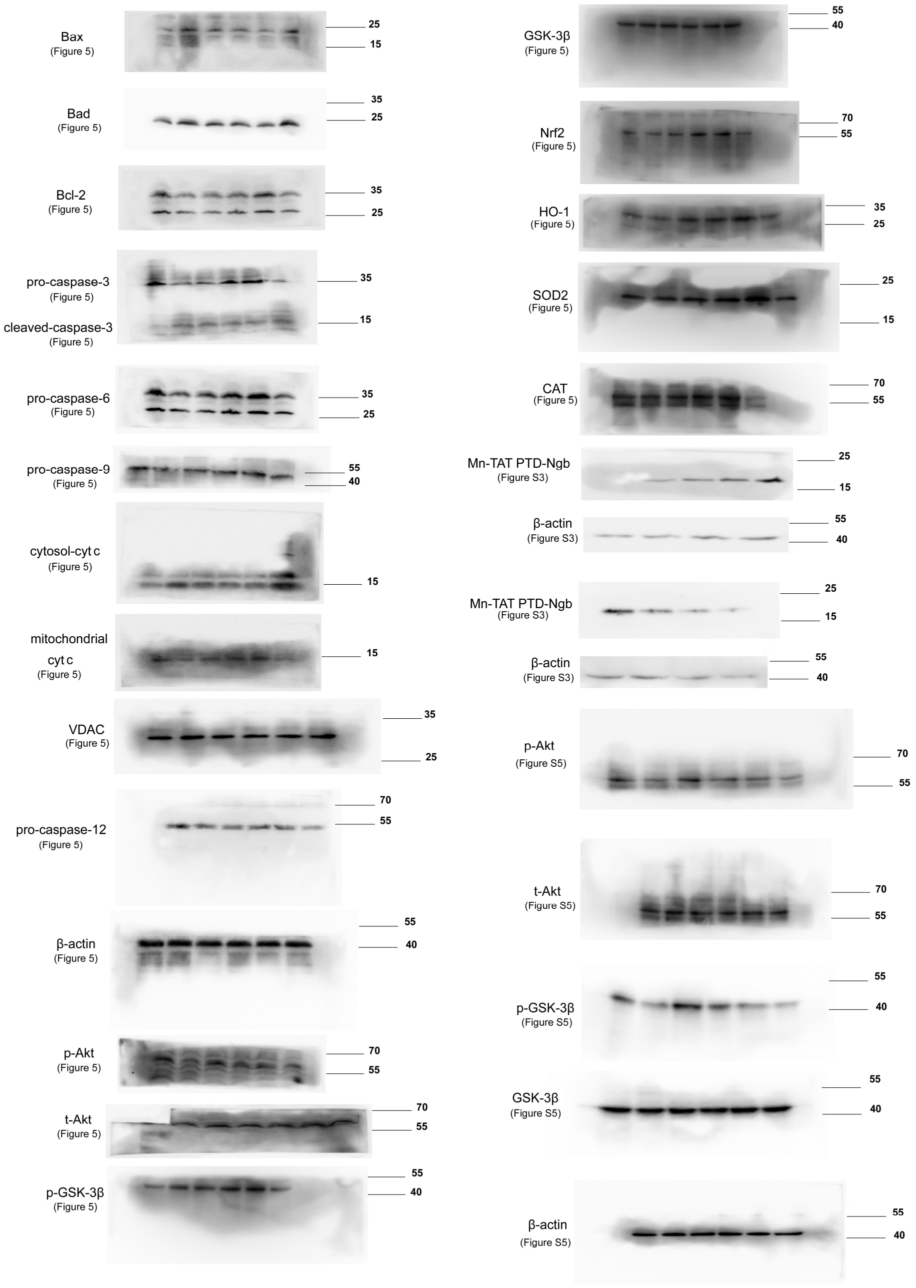

Fig. S6. Full length blots of the cropped Bax, Bad, Bcl-2, Caspase-3, Caspase-6, Caspase-9, cytochrome c in mitochondrial and cytosol fraction, Caspase-12, β-actin, p-Akt, t-Akt, p-GSK-3β, GSK-3β, Nrf2, HO-1, SOD2, CAT, Anti-6×His-tag presented in the main text. The primary antibodies directed to Caspase-3 (1:1000, CST), Caspase-9 (1:1000, CST), Caspase-6 (1:1000, CST), Caspase-12 (1:1000, CST), Bcl-2 (1:1000, CST), β-actin (1:1000, Beyotime, No. AA128), Bad (1:1000, CST), Bax (1:1000, CST), Cyt c (1:1000, CST), Anti-6×His-tag (1:1000, Sangon Biotech), GSK-3β (1:1000, CST), p-GSK-3β (1:1000, CST), SOD2 (1:1000, CST) t-Akt (1:1000, ImmunoWay), p-Akt (1:1000, ImmunoWay), Nrf2 (1:1000, Abcam), CAT (1:1000, Absin), HO-1 (1:1000, Absin) were used. The

samples were derived from the same experiment and the blots were processed in parallel. The exposure time of each blot was 10 s.
